# Non-Intrusive Assessment of COVID-19 Lockdown Follow-Up and Impact Using Credit Card Information: Case Study in Chile

**DOI:** 10.3390/ijerph18115507

**Published:** 2021-05-21

**Authors:** Ricardo Muñoz-Cancino, Sebastian A. Rios, Marcel Goic, Manuel Graña

**Affiliations:** 1 Business Intelligence Research Center (CEINE), Department of Industrial Engineering, University of Chile, Beauchef 851, Santiago 8370456, Chile; srios@dii.uchile.cl; 2 Department of Industrial Engineering, University of Chile, Beauchef 851, Santiago 8370456, Chile; mgoic@dii.uchile.cl; 3 Computational Intelligence Group, University of Basque Country, 20018 San Sebastian, Spain; manuel.grana@ehu.eus

**Keywords:** COVID-19, topic modeling, credit card data, economic impact of lockdown measures

## Abstract

In this paper, we propose and validate with data extracted from the city of Santiago, capital of Chile, a methodology to assess the actual impact of lockdown measures based on the anonymized and geolocated data from credit card transactions. Using unsupervised Latent Dirichlet Allocation (LDA) semantic topic discovery, we identify temporal patterns in the use of credit cards that allow us to quantitatively assess the changes in the behavior of the people under the lockdown measures because of the COVID-19 pandemic. An unsupervised latent topic analysis uncovers the main patterns of credit card transaction activity that explain the behavior of the inhabitants of Santiago City. The approach is non-intrusive because it does not require the collaboration of people for providing the anonymous data. It does not interfere with the actual behavior of the people in the city; hence, it does not introduce any bias. We identify a strong downturn of the economic activity as measured by credit card transactions (down to 70%), and thus of the economic activity, in city sections (communes) that were subjected to lockdown versus communes without lockdown. This change in behavior is confirmed by independent data from mobile phone connectivity. The reduction of activity emerges before the actual lockdowns were enforced, suggesting that the population was spontaneously implementing the required measures for slowing virus propagation.

## 1. Introduction

Since the early days of 2020, the COVID-19 pandemic has been shocking [1] the world [2,3], with several waves of the COVID-19 outbreak hitting differently in all countries [4], even showing different incidence inside the administrative partitions of the countries [5]. The main tools that have been proposed [6] to control the damage of a viral infection outbreak are related to either non-pharmacological interventions (NPI) impeding the spread of the virus or pharmacological interventions aiming to treat the associated disease severity. Regarding pharmacological developments, there are many studies trying to assess the benefits of several ancient and new molecules against the SARS-CoV-2 virus [7,8,9,10], while vaccinal approaches are being tested at large on the world population [11]. Regarding non-pharmacological interventions [12,13,14], many countries or their lower administrative divisions (such as states, regions, or counties) have implemented quarantines and other restrictions of movement, while recommending social distancing, wearing masks and general prophylaxis measures. Concurrently, there is growing concern about the side effects of these measures on the general and at-risk population, specifically children and young adults [15,16,17,18]. For instance, the use of urban green spaces has been greatly affected by the pandemic, and it has been valued highly as a resource to overcome the mental burden of the situation [19]. Specifically, the Chilean government enforced several NPI steps in order to reduce the health risk impact of COVID-19. On 16 March 2020, all schools were closed, and, in the same week, workplaces implemented work-from-home strategies. On 17 March, all national parks were closed, and from 26 March until the time of writing this paper, lockdowns are being enforced on districts (communes) with COVID cases above a given threshold. Lifting the lockdown is progressive in several steps. A curfew from 21:00 until 5:00 is enacted each night over all the territory. The real-time situation is published on the following website https://www.gob.cl/coronavirus/cifrasoficiales/ (accessed on 20 May 2021).

In this paper, we consider transactions from credit and debit cards (Credit Card Records—CCR) for the assessment of the change in behavior of the population during the implementation of the lockdown measures. After appropriate anonymization, CCR provide high-resolution spatiotemporal information on the economic life of the city. The geo-location of credit card transactions is achieved through the Point of Sale Terminal (POST) where the transaction is made, which provides additional information about the economic sector involved in the transaction.

There is literature emerging about the observation of the economic impact of COVID-19 through the lens of consumer spending obtained from CCR information. For instance, CCR data from the second biggest bank in Spain shows a sharp v-shape in the aggregated consumption due to the strict lockdown measures imposed by the Spanish government [20], while the authors of [1] used the aggregate information in a predictive epidemiological model of pandemic evolution in Turkey. A detailed study over the period March–August 2020 on the USA credit card market [21] found a sharp decrease in transactions and balances in mid-March with a slow incomplete recovery from May onwards. Some economic sectors did experience sharp decreases in activity while others actually increased the volume of transactions [22]. The study in [21] compares the effect of NPI measures with the psychological pressure of the pandemic roughly measured by the number of cases in the surrounding areas, also known as pandemic severity, finding that pandemic severity has a stronger effect on the diminishing credit card transactions. Pandemic fatigue implies that this effect is weakening over time since the pandemic outburst. The effect of pandemic severity over the volume of consumer transactions has also been observed in China since the pandemic outbreak in January 2020 [23]. A similar v-shape decrease in volume of transactions was found in France, where the decrease in credit card transactions started a couple of weeks before the lockdown measures [24]. An additional confirmation of the separate effects of pandemic severity and mandated NPI on consumer spending is the comparison of the volume of all means of electronic transactions in Sweden and Denmark during the early months of 2020 [25].

However, little research at a microscopic level has been done about the impact on consumer behavior of stay-at-home mandates and other NPI measures. A relevant research question addressed in this paper is whether the NPI anti-COVID-19 measures have induced behavioral changes in the spending habits of the inhabitants of Santiago. We use results from independent studies based on cell-phone calls (Call Detail Record—CDR) to confirm/validate our results [26,27]. Additionally, we consider whether the lockdown measures have induced a downturn in the flow of economic transactions that may have a long-term effect on the quality of life of city areas most affected, with a potential health impact on the future. Specifically, the appearance of decreasing economic activity before the enforcement of NPI should be a confirmation that pandemic severity perception by the public is a separate strong effect of socio/economic activity.

## 2. Materials and Methods

### 2.1. Geographical Location and Scope

**Figure 1:**
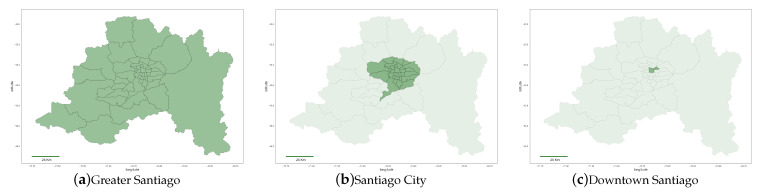
Santiago Metropolitan Region.

In this paper, we deal with data from the city of Santiago, Chile, which can be defined at three geographic levels. Administratively, the city of Santiago is organized into communes, the borders of which are shown in Figure 1a, with a high degree of autonomy. First, we may consider the Metropolitan Region where the city of Santiago is located. The Metropolitan Region has a population of almost 7 million inhabitants and a total area of 15,400 km${}^{2}$. Second, we may consider Santiago City itself, which is a smaller area of the greater Metropolitan Region that excludes a number of rural areas. Finally, we may consider Santiago downtown, where a large fraction of business offices are concentrated, corresponding to the two most crowded communes of Santiago. These three areas are illustrated in Figure 1. In this paper, we focus on the analysis of the data relative to Santiago City because it is where most of the economic activity concentrates (Figure 1b).

### 2.2. Credit Card Data

Computational experiments were carried out over an anonymized dataset provided by a major Chilean bank collected under strict personal data protection protocols. The study has been approved by the Ethics and Biosecurity Committee of the Facultad de Ciencias Físicas y Matemáticas of the University of Chile. Before being used in this study, customer identifiers, credit card identifiers, and any personal data were removed before entering the data analysis described below. There is no possibility that personal information can be leaked and no result of our analysis can be associated to any particular cardholder because the analysis is only based on the number of transactions made in every time window at each business location.

A Point of Sale Terminal (POST) is an electronic device that is used to process card payments at business locations. Each time a customer pays with a credit or debit card, the transaction is processed by the POST and is geographically tagged with the latitude and longitude of the associated business location. Each record in the dataset contains the card type (credit or debit), ID, latitude and longitude of POST where the transaction was made, day and hour of the transaction. Furthermore, the record contains the economic sector related to the company where the transaction was made. The dataset contains credit card transactions made during the years 2017, 2019, and 2020 associated with more than 100 thousand POSTs. Specifically, we have 85, 60, and 45 million credit card transactions for the years 2017, 2019, and 2020, respectively.

A POST Activity Pattern (AP) is a vector characterizing the activity of each terminal *p* over a specified period of time. In this study, we will consider that each AP describes the weekly activity of the POST. Formally speaking, we describe a raw activity pattern by a vector $\mathrm{XP}{}^{p}$, where each component $XP_{t}^{p}$ (activity block) denotes the number of transactions carried out on terminal *p* during period *t*. Then, the raw activity pattern of the terminal *p* is given by the vector $\mathrm{XP}{}^{p}=\left(XP_{1}^{p},XP_{2}^{p},\ldots ,XP_{T}^{p}\right)\left(t\in T\right)$, where *T* is the set of activity blocks. In this study, we set *T* into hourly periods during a week; therefore, each raw activity pattern $XP^{p}$ is a vector with card(T)=168 components (24 h, seven days). To facilitate the comparison between terminals, in the extraction of the $\mathrm{XP}{}^{p}$ from the bare dataset of card transactions, we define the normalized activity pattern $\mathrm{AP}{}^{p}$, where $AP_{t}^{p}=\frac{XP_{t}^{p}}{\sum_{t\in T}XP_{t}^{p}}$, i.e., dividing its components by the total number of transactions of each terminal. Therefore, we can interpret $AP_{t}^{p}$ as the percentage of transactions of the terminal *p* processed in the hourly time window *t*.

**Figure 2:**
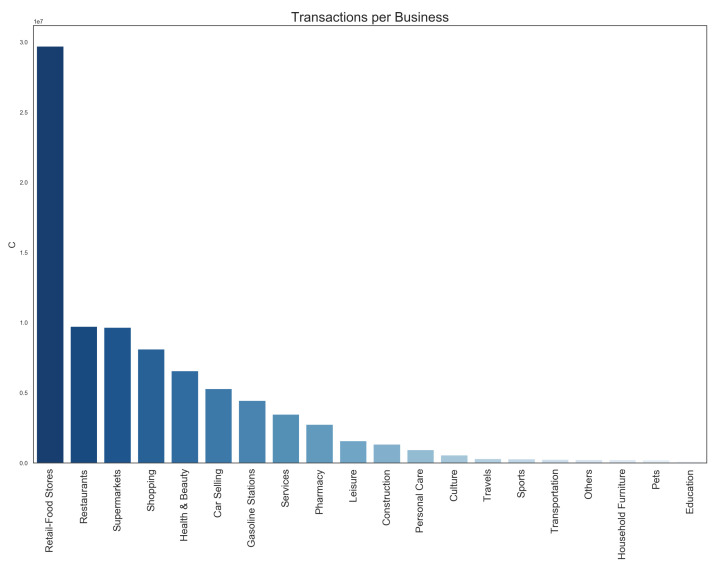
Distribution of the amount of CCR transactions per economic sector during the entire year of 2017.

Using the economic sector information of each POST, we can determine the distribution of card usage over the economic sectors, as shown in Figure 2, for the entire year 2017. Here, we observe that the retail food stores represent almost 35% of the transactions in a normal year, followed by restaurants and supermarkets, each with a share of 10% of the volume of transactions. Due to the high frequency of the transactions done in these businesses, they will provide detailed information on the spatial distribution of economic transactions. Notice that relative small number of transactions in transportation or education do not imply that CCR payment data is blind to these activities. For example, if a student purchases a meal nearby her college campus, we will count that transaction, and we will assign it to a cell that is close to the corresponding campus.

### 2.3. Spatial Aggregation

**Figure 3:**
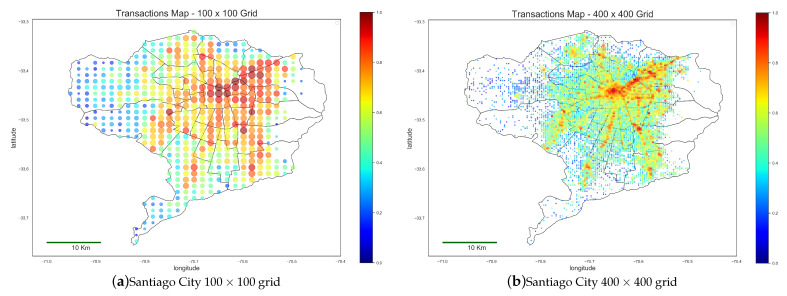
Spatial aggregation grids over Santiago City.

In order to visualize the geographical distribution of the volume of transactions, we carry out a spatial aggregation that is the result of imposing upon the city a grid of n×n uniform cells (it is well known [28] that hexagonal tiles provide a more precise tessellation of the plane, but for our purposes, the ease of applying square cells overcomes the loss of precision.). Each cell will be represented by a single AP that is the average of the AP of the POSTs falling inside the cell. Figure 3 illustrates two spatial aggregation grids over Santiago city (a 100 × 100 grid (10,000 cells) and a 400 × 400 grid (160,000 cells)), showing in logarithmic scale the total number of credit card transactions made inside each cell.

Under this spatial aggregation scheme, we define $\mathrm{XP}{}_{i,j}$ as the raw activity pattern for the cell (i,j) in the n×n grid (i=1,...,n and j=1,...,n) as:

(1) $$\mathrm{XP}{}_{i,j}=\frac{1}{\mathrm{card}(H_{i,j})}\sum_{h\in H_{i,j}}{\mathrm{XP}}^{h}$$

where $H_{i,j}$ is the set of POSTs inside the cell (i,j). Each cell (i,j) is characterized by the total number of transactions made in the cell (i,j) and the total number of POSTs in the cell, denoted $\mathrm{card}(H_{i,j})$. In the same way, $\mathrm{AP}{}_{i,j}$ denotes the normalized activity pattern in the cell:

(2) $$\mathrm{AP}{}_{i,j}=\frac{1}{\mathrm{card}(H_{i,j})}\sum_{h\in H_{i,j}}{\mathrm{AP}}^{h}$$

**Figure 4:**
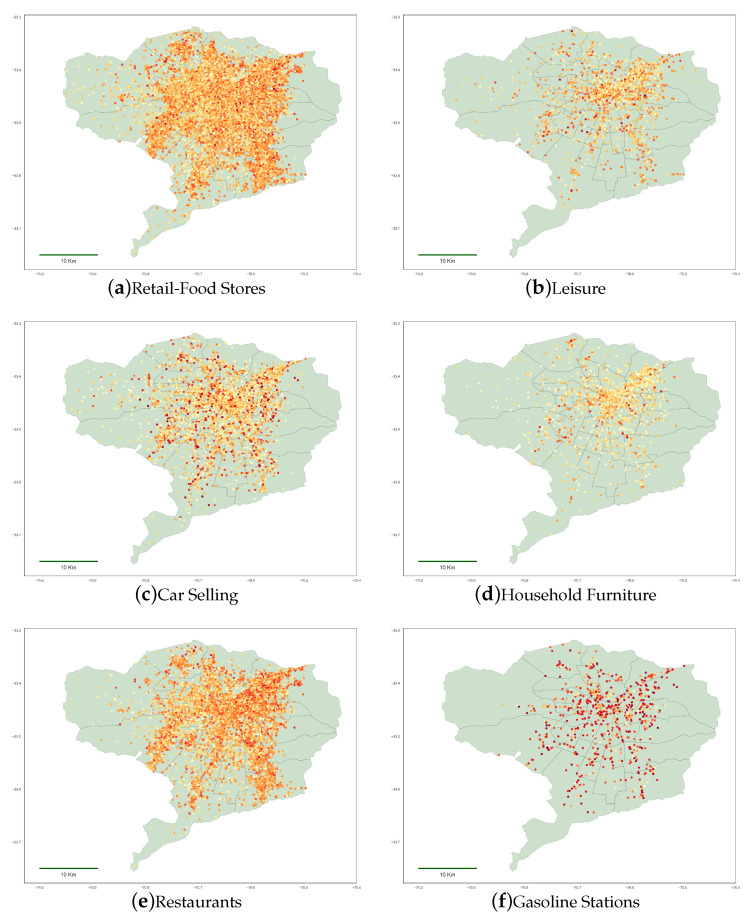
Spatial distribution of credit card transactions per POST and economic sector during the year of 2017 in Santiago City.

To better illustrate how different economic sectors are geographically distributed in the area under study, in Figure 4, we show the number of transactions per POST for a selected number of economic sectors in Santiago City. The uneven distribution through different economic sectors in different areas of the city illustrates the ability of CCR to inform spatiotemporal economic patterns in urban areas.

### 2.4. Determination of Activity Pattern Duration

**Figure 5:**
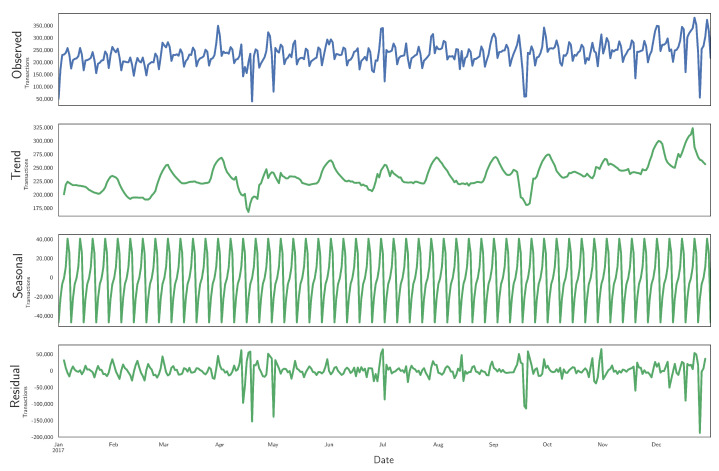
Decomposition of the aggregated CCR time series into trend, seasonal, and residual components.

Figure 5 displays the time series of the number of transactions (upper plot) along with a seasonal decomposition using an additive model from the python library statsmodels (https://www.statsmodels.org/stable/generated/statsmodels.tsa.seasonal.seasonal_decompose.html) (accessed on 20 May 2021). The trend is obtained by applying a convolutional filter that implements a moving average, and the seasonal component corresponds to the average for each period of the de-trended series. The trend component shows an increment at the end of each month, explained because a significant fraction of Chilean citizens is paid precisely at the end of the month. The trend also shows a moderate increment by the end of the year, associated with Christmas shopping. The seasonal component has a strong weekly frequency. Based on this weekly regularity, we define APs to have a week duration. In other words, one week is our time frame to describe the activity at each terminal and to extract representative patterns (topics) of the distribution of transactions for a typical year.

### 2.5. Topic Modeling of CCR Data

Topic modeling research originates from natural language processing of documents. It is a probabilistic approach for discovering latent topics in a collection of documents. The basic premise is that the words that compose the documents are a linear combination of these latent topics. The most successful technique for topic modeling is the Latent Dirichlet Allocation (LDA) [29]. In this work, we apply this approach to characterize human activities in the city using geographically tagged credit and debit card transaction data. Essentially, topic modeling assumes that any activity pattern ${\mathrm{AP}}^{p}$ of a POST can be expressed as a linear combination of *K* activity topics $\left\{\mathrm{AT}{}^{0},\ldots ,\mathrm{AT}{}^{K-1}\right\}$, i.e., ${\mathrm{AP}}^{p}=\sum_{k=0}^{K-1}\theta_{k}^{p}\mathrm{AT}{}^{k}$. Thus, each POST activity pattern ${\mathrm{AP}}^{p}$ is described by a mixing of activity topics $\theta^{p}$, also known as topic distribution of the document. The generative model of LDA assumes that $\theta^{p}$ follows a Dirichlet distribution of symmetric parameter α<1. Activity blocks are the equivalent in our problem to the words in the document processing applications, i.e., the possible values of activity at each hour $A={\left\{AP_{t}^{p}\right\}}_{t,p},$ where *t* and *p* extend over the hours in the week and all POSTs, respectively, without duplicated values. Activity topics are composed of activity blocks, with mixing parameters $\varphi^{k}$ that follow another Dirichlet distribution of a symmetric parameter β<1. The topic that the activity block $AP_{t}^{p}$ belongs to is denoted by $z_{tp}$, which follows a multinomial distribution of parameters $\theta^{p}$. Finally, the activity block $AP_{t}^{p}$ in each time position *t* of the activity pattern ${\mathrm{AP}}^{p}$ follows a multinomial distribution of parameters $\varphi^{z_{tp}}$. The LDA generative model proceeds by the generation of the topics in the document (activity pattern), the words (activity blocks) in each document, the precise topic for each word, and the selection of the words in each position of the document.

To build up the LDA model, i.e., to discover the latent activity topics and the decomposition of the POST activity patterns into them, we used a python implementation provided by gemsim (https://radimrehurek.com/gensim/) (accessed on 20 May 2021). The LDA discovery of latent topics is unsupervised; therefore, there is no guarantee regarding the order of discovery or the identity of the topics. In order to establish correspondences among topics in different sets, e.g., discovered from data extracted at different times, we use the cosine similarity metric between two activity patterns (AP) defined as follows:

$$\begin{array}{ccc} COS\left(AP_{b},AP_{c}\right)=\frac{\sum_{i=1..T}A_{i}^{b}\cdot A_{i}^{c}}{\sqrt{\sum_{i=1..T}{\left(A_{i}^{b}\right)}^{2}}\cdot \sqrt{\sum_{i=1..T}{\left(A_{i}^{c}\right)}^{2}}} & & . \end{array}$$

The cosine similarity value is in the range [−1,1] and equals 1 only when the two activity patterns, $AP_{b}$ and $AP_{c}$, exactly coincide. It is a measure of the relative orientation of the high dimensional vectors, thus very insensitive to the absolute magnitude of vector components and equivalent to the correlation measure for zero mean vectors. The cosine similarity has been extensively used in studies about geographical distribution of land uses [30] using diverse information sources, such as Twitter activity [31] and Flickr tags [32].

## 3. Results

### 3.1. CCR Activity Topics before the Pandemic

**Figure 6:**
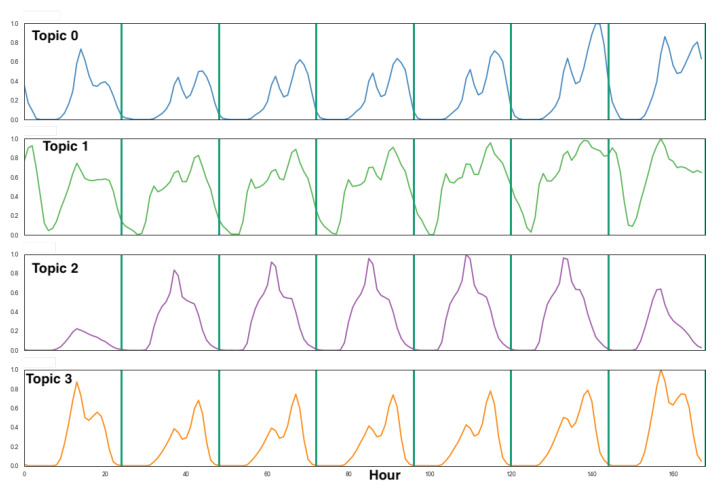
LDA detected topics in Santiago City, K=4. The vertical partitions correspond to days of the week starting from Sunday. The *x*-axis is the time measured in hours. The *y*-axis is the normalized values of the activity pattern.

In this study, we first process the CCR data during the year 2017, well before the pandemic, in order to obtain reference CCR activity topics extracted by the LDA algorithm. To set the value of parameter *K*, we carried out an exploration of LDA results for each value of K∈{2,…,6}, and selected the value of K=4, which maximizes the information content and the interpretability of the CCR activity topics. We measure the information content of the set of extracted topics by their direction divergence, i.e., more divergent vectors provide better representation axes to describe the space of vectors under analysis, in this case, POST activity patterns. Therefore, we compute the cosine similarities between all possible pairs of activity topics, using the norm of the resulting matrix as the information measure that we want to minimize. In all exploration experiments, we found that K=4 provided a minimum value of this information measure relative to other selections of *K*. Figure 6 shows the optimal CCR activity topics obtained for the area of Santiago City. The vertical partitions correspond to days of the week starting from Sunday. The *x*-axis is the time measured in hours. The *y*-axis is the normalized values of the activity pattern computed dividing all vector components by the value of the maximum component. Overall, these patterns are consistent with those reported on previous work using other data sources (see, for example, [26,33]).

The interpretation of these topics is as follows:

- Topic 0 “residential” is characterized by two high activity peaks during weekdays, localized around lunch and dinner times. Notice that the second peak on Fridays occurs later around 11 p.m., reflecting that people used to have a later dinner on this day. Also notice that the second peak on Sundays is much less pronounced, indicating that citizens were less prone to go out dining on Sunday evening.
- Topic 1 “leisure/commerce” presents three peaks during the weekdays located at 9 a.m., lunchtime, and around 7 p.m., roughly corresponding to times when people used to commute to or from school or work. During weekends, this pattern is more evenly distributed throughout the day. Notice that there are high activities in the early hours of Saturday and Sunday, corresponding to nightlife habits before the pandemic.
- Topic 2 “office areas” is characterized by a high and uniform activity during weekdays and less activity during weekends. During the day the main activity is between 09:00 a.m. and 18:00 p.m. and there is increasing activity at lunchtime (13:00 p.m.), corresponding to office areas activity.
- Topic 3 “rush hour” has peaks that roughly correspond to times when people are moving from or to the working place in office/business areas.

For further confirmation of the above interpretation of the CCR activity topics, we compare them with CDR activity topics reported in previous analyses of human activity patterns in Santiago [26]. The results for the cosine similarity between the activity topics discovered using CCR and CDR data are shown in Table 1. These results show that the discovered activity topics were relatively stable before the pandemic, independent from the data source and time frame. Indeed, every topic detected from CCR data is associated with a topic detected from CDR data with a large cosine similarity. The interpretation of the CDR activity topics [26] is similar to the one above for CCR data. We find cosine similarity above 0.8 for rush hour and residential, and above 0.93 for leisure/commerce and office areas, respectively.

**Figure 7:**
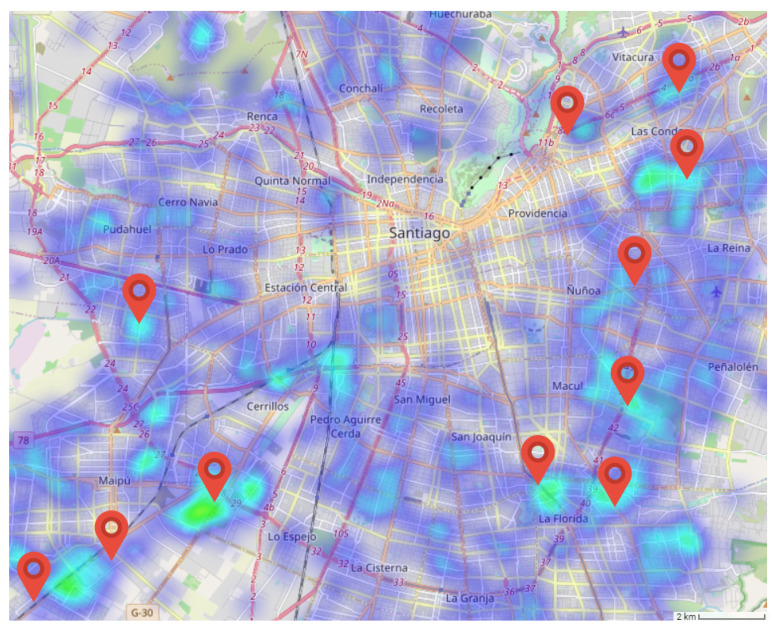
Spatial distribution of the leisure/commerce CCR activity topic, overlaid by the localization of the main shopping malls. Blue color blobs spot the localization of POSTs with high contribution of this activity topic in their LDA decomposition.

Additional confirmation of our interpretation of the CCR activity topics comes from the observation of the spatial distribution of the topic in the area of Santiago. Recall that LDA’s output can be interpreted as the degree $g_{k,p}$ that each activity topic *k* contributes to the overall activity pattern of POST *p*, such that $\sum_{k}g_{k,p}=1\;\forall p$. Hence, we compute the contribution of each activity topic to the aggregated activity of each spatial cell $g_{k,\left(i,j\right)}$. Figure 7 displays the spatial distribution of the leisure/commerce activity topic in the area of Santiago City overlaid with the exact location of the major shopping malls in Santiago. It can be appreciated that they correlate well, confirming the interpretation given above. We focus on this topic because it reflects the greatest changes under the lockdown measures for contention of the COVID-19 pandemic.

### 3.2. Change in Activity Topics Due to the Pandemic

**Figure 8:**
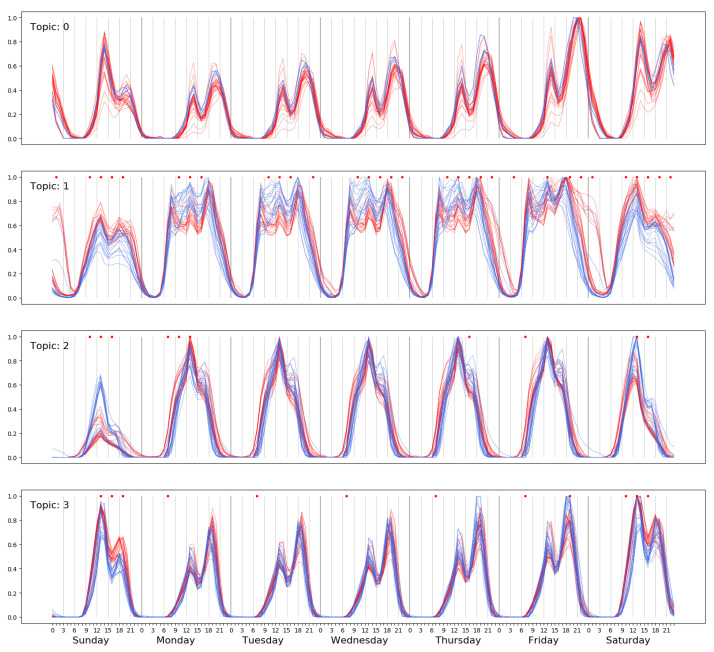
Change in activity topics due to the lockdowns and curfews imposed to curb the pandemic. Dark blue and red lines correspond to topics extracted from data of 2020 and 2019, respectively. The red dots denote statistically significant (*p* < 0.0001) differences among pre-pandemic and pandemic activity topics in aggregations of 3 h.

In order to assess the effect of NPI for COVID-19 on the behavior of the habitants of Santiago, we collected the CCR data in pre-pandemic (year 2019) and pandemic (year 2020) periods. We remind the reader that Chile has been in commune-based lockdown ever since mid-March 2020, so there is no global post-lockdown period. In order to have a picture of the evolution of the activity topics, we proceed to extract the LDA topics (K=4) of activity patterns from windows of 12 consecutive weeks, with an overlap of 10 weeks between consecutive windows. Therefore, we have 13 sets of LDA activity topics per year. Figure 8 shows the overlaid activity topics of year 2019 (red) and year 2020 (dark blue). In order to have similar topic assignation, we compute the cosine distance of the LDA detected topics on a 12-week window against the topics extracted from data of the year 2017 described above. We assign 2017 topics to the topics discovered in the time windows of 2019 and 2020, which are more similar according to the cosine distance, so that the meaning of the topics remains constant. Thus, Topic 1 always corresponds to the leisure/commerce activity topic, which has been most strongly affected by lockdown and curfews. It can be appreciated that the late-night expenditures during the weekend have disappeared (red arrow). For a more quantitative appraisal of the changes between the activity topics assigned to the leisure/commerce from years 2019 and 2020, we aggregate 3 h intervals and compute a two-sided non-parametric Wilkinson test to assess the statistical significance of the differences of activity. We have highlighted with a red star those 3-hour periods that have strong significant differences (p<0.0001). The strong impact that the non-pharmaceutical interventions have had on the behavior of the citizens of Santiago City can be appreciated.

### 3.3. Impact of Local Policies

**Figure 9:**
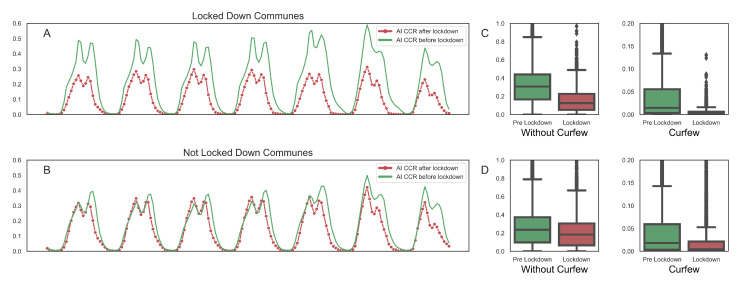
The effect of lockdown and curfew policies in Santiago, Chile. (**A**) Average weekly activity before and after lockdown for communes enforcing lockdown. (**B**) The same for communes not enforcing lockdown. (**C**) Additional impact of curfew on communes that enforced lockdown. (**D**) The same for communes that did not enforce lockdown.

The observation of the overall economic activity previous to the declaration of lockdowns and curfews allows to assess their actual implementation and impact. Figure 9 displays the weekly activity pattern inferred from payment data with and without mobility restrictions. Considering that the contagion dynamics of the pandemic are not strongly related to the specific activity topic of each terminal, we display aggregated measures regardless of the underlying topics, i.e., the average hourly activity. Figure 9A plots the average activity before (green) and after (red) lockdown in communes that did implement lockdown policies, showing a significant decrease in activity. If additionally, we consider the implementation of curfew for these communes, the box plots in Figure 9C show a huge drop in economic activity after the curfew was declared. Communes that did not implement lockdown were less affected, as shown in Figure 9B. Nevertheless, implementation of curfews had a significant impact for them, as shown by the box plots of Figure 9D.

### 3.4. Aggregate Activity Measurement of Impact

**Figure 10:**
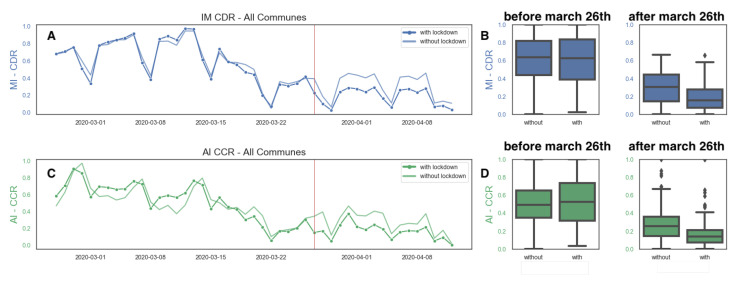
The effect of lockdown policies in Santiago, Chile. Aggregated data from the beginning of March 2020 until April 15th. The red line indicates March 26th. (**A**) CDR activity for communes with and without lockdown. (**B**) Box-plots of CDR activity in communes with and without lockdown before and after March 26th. (**C**) CCR activity for communes with and without lockdown. (**D**) Box plots of CCR activity in communes with and without lockdown before and after March 26th.

To assess the impact of non-pharmaceutical interventions implemented to curb the COVID-19 pandemic, we are also interested in the overall change in activity inferred from CCR data, and how it compares with changes in activity inferred from CDR data [27]. We compute the overall daily activity levels from both data sources for communes that have implemented lockdown and those that have not, as illustrated in Figure 10, since the beginning of the month of March until mid-April. The critical date of March 26th is highlighted by a red line. We can appreciate in both Figure 10B,D that there is a sharp decrease of activity in both CDR and CCR data for all communes regardless of their implementation of lockdowns. Furthermore, it can be appreciated in Figure 10A,C that there is a sharp slowdown of activity in both CDR and CCR data almost ten days before the decision to implement lockdowns. Consistent with the early evidence presented in other countries [34], a relevant reduction in mobility and economic activity was voluntarily adopted for many citizens, but the lockdown policy generated an additional impact.

In spite of some variations, the overall trend captured by both data sources is very consistent, and they are associated with similar estimates in the reduction of activity due to the lockdown policy, as shown in Table 2. Using CCR data we estimate a reduction of 45.9% for communes not directly affected by the lockdowns and a 50.7% reduction if we estimate it using CDR data. Similarly, for communes that were implementing a mandatory lockdown, the reduction in activity from both sources is almost identical, with 69.6% for the CCR estimate and 69.7% with the CRR data. This agreement between data sources comes as a validation of the use of CCR data for the assessment of the implementation of non-pharmaceutical interventions.

## 4. Discussion

Regarding the assessment of the impact of non-pharmaceutical interventions against the COVID-19 pandemic put in place by the Chilean government, CCR data analysis can provide information about the mobility of the population and the effect on the economic sectors of the changes of population mobility. In this regard, we used CCR data to evaluate if the impact in mobility of the adoption of stay-at-home policies that encourage individuals to reduce non-essential trips has been reflected in changes in economic activities. Furthermore, we have been able compare the estimates in mobility against those derived from the CDR data in the period of time analyzed [27]. Other approaches to assess the economic impact of the pandemic use electric consumption as a proxy [35].

The analysis of the CCR data identifies an economic sector that has hardly been hit by the pandemic-induced economic crisis, that of Leisure/Commerce out of the four activity topics identified on the pre-pandemic data. Our results comparing this activity topic in the year previous to the pandemic and the year of the pandemic show that Santiago citizens have changed their behaviors according to the lockdown and curfew policies. Though there is some literature on the disruption of supply chains due to COVID-19 [36], the impact on healthcare [37], forest degradation [38], the economic impact in African countries (e.g., the impact on cattle exports) [39], and on residents of some cities [40], there is little regarding the evaluation of the impact of COVID-19 on the people that work in the leisure/commerce sector in developed countries, except for some high-level analysis at the corporate level [41,42]. However, there is a large percentage of the labor force enrolled in the leisure and hospitality sector (i.e., more than 13 million employees in March 2021 in the US, according to workforce statistics https://www.bls.gov/iag/tgs/iag70.htm#iag70emp1.f.p (accessed on 20 May 2021)) that may fall into poverty with ensuing systemic health critical issues.

One of the facts that we have found is the voluntary reduction of activity that was apparent several days before the implementation of coercive measures from the administrations. This result is in agreement with reported voluntary reductions in mobility estimated from the Google human mobility dataset [43]. It seems that accurate information about the evolution of the pandemic helps the citizens to make appropriate decisions toward curbing the pandemic. How the required reduction of mobility impacts the economic activity is not clear, as some researchers argue that increasing activity in parks and groceries/pharmacies has much less effect on the reproductive rate than staying at home [44]. Travel patterns appear to have a huge impact in the propagation of the virus, requiring a combination of sensible public policies and the willing collaboration of the community, as demonstrated by the case of Hong Kong [45]. Big data extensive studies have found that imposed public policies play a small role in the reduction of mobility [46]. In fact, the main factor contributing to reductions in mobility appears to be the fear of contagion [47]. Mobility and its relation to economic activity in times of a pandemic need a further research agenda [48].

## 5. Conclusions

This paper shows how the anonymized information about credit card transactions can be used to assess the follow-up and impact of non-pharmaceutical interventions implemented to curb the COVID-19 pandemic. We show how unsupervised latent topic analysis uncovers the main patterns of credit card transaction activity that explain the behavior of the inhabitants of Santiago City. Topics identified in the pre-pandemic year of 2017 are used to identify the topics produced by the analysis in years 2019–2020, including the pandemic. Specifically, we are able to assess the impact on the leisure/commerce sector, which has suffered a strong loss of activity due to the pandemic. Additionally, the examination of the aggregated activity allows assessing significant differences among communes that did impose lockdown from those that did not. Lockdown and curfew interventions lead to a reduction of 70% in credit card transaction activity. Additionally, we found a spontaneous reduction of activity before the implementation of lockdown of the same magnitude of the reduction achieved with the mandatory restrictions. The need for coercive measures to achieve mobility reduction to stop the virus spread may be reexamined in light of these findings. Future works will be directed to the disaggregate analysis of the information of the points of sale according to their nominal industrial activity, in order to ascertain the variable impact of the pandemic on the industry. This analysis will include the distinction between essential and non-essential services. In addition, a detailed analysis of the recovery after lockdown should be carried out independently for each commune.

## Figures and Tables

**Table 1:** Cosine similarity between topics discovered over CCR and CDR data [26] in the Santiago City area. Bold numbers are maximal values per column.

		CDR			
		T0	T1	T2	T3
CCR	T0	0.63	**0.82**	0.72	0.42
	T1	**0.80**	0.76	0.76	0.70
	T2	0.60	0.41	0.59	**0.93**
	T3	0.69	0.63	**0.94**	0.56

**Table 2:** The overall effect of lockdown policies in Santiago, Chile, measured from mobile phone connectivity (CDR) and credit card transactions (CCR).

Data Source	Lockdown Commune	Before March 26th	After March 26th	Reduction (%)
CDR	No	0.614	0.303	50.7%
CDR	Yes	0.604	0.183	69.7%
CCR	No	0.503	0.272	45.9%
CCR	Yes	0.520	0.158	69.6%

## Data Availability

Data are proprietary. Access to the data needs the review and consent of the owner.
